# Keeping it real: A journal editor in clinic

**DOI:** 10.1371/journal.pmed.1002394

**Published:** 2017-09-26

**Authors:** Larry Peiperl, on behalf of The PLOS Medicine Editors

**Affiliations:** Public Library of Science, San Francisco, California, United States of America and Cambridge, United Kingdom

## Abstract

In this month’s editorial, *PLOS Medicine*’s Chief Editor Larry Peiperl discusses the relevance of patient care to a journal editor’s work.

Providing conscientious care despite imperfect knowledge and limited resources: this has become a realistic expectation for many physicians, in high-income as well as lower-income countries. “Half of what we teach you will turn out to be wrong,” a professor warned my class of new medical students almost 30 years ago; “We just don’t know which half.” Seven years later, our chair of Internal Medicine quoted Yogi Berra to senior residents who were about to disperse into the early years of managed care in the United States: “The future is not what it used to be.” We learned not only that our evidence base was subject to question, but that it would also be our role to support patients through systems of care that could at times defy even the soundest clinical reasoning. Clinical practice would remain, as expressed in our hospital’s unofficial motto, “As real as it gets” [1].

Editing a medical journal has its own challenges, not least of which is having to decide which advances are likely to be of practical interest. In an ideal world, every willing physician would spend some time at the office of a medical journal. Active practitioners could tell editors directly what makes clinical research interesting or useful. Many would be interested to see the processes by which research submissions become “the literature”—the term by which physicians dignify the accumulated knowledge in their area of practice. General medical journals are relatively few, however, and practitioners are many. To ensure that journals remain engaged in the realities of medical practice, it makes more sense for physicians who become editors to spend time in patient care.

The editor of a prominent medical journal reminded me of this at a dinner meeting not long ago. I mentioned that it had been a couple of years since I’d worked regularly in a clinic because of the pressures of editorial work. He looked at me sharply and pointed out that my perspectives were at risk of becoming irrelevant. Nearby editors of other journals nodded their assent. Faintly protesting that I’d just passed my specialty board’s recertification exam, I reflected that they were probably right. I tried to tell myself that taking good care of manuscripts might eventually benefit more patients than I could ever help personally, yet during editorial meetings I would often slip and say “this patient” instead of “this paper,” to the amusement of my colleagues. In this role of impartial appraisal I missed warming up my hands to examine the carotid pulse, or encouraging an intern to sit at the patient’s eye level for a difficult conversation. I missed supporting the leading role that the body plays, sooner or later, in each of our lives.

While the responsibilities of editor and physician are hardly interchangeable, parallels do exist. The main role of a medical journal editor is to tend the evidence base. Usually this means ensuring, largely through watchfully managed peer review, the validity and transparency of articles that are to enter “the literature.” Toning down exuberant claims, identifying methodological biases, remaining alert to issues of ethics and confidentiality—for editors, these are as sore throats and low back pain are to the clinician: collectively routine but individually unique and requiring vigilance for the few cases that may represent a more serious problem.

The main role of a physician is to attend the patient. Usually this means mediating between the generalizations of published research and the specific circumstances and wishes of the individual. The clinician’s challenge is to keep up with the literature, discern the relevant aspects of the patient’s story, and propose a plan that aligns them as effectively as the available knowledge and resources permit, all the while recognizing when to respectfully question the literature, the story, or—when costs come into conflict with clinical benefit—the available resources.

I feel fortunate to have recently been reappointed to the medical staff of the public hospital where I trained in primary care and where I have seen patients and taught over many years. About once a week, I work as preceptor—a teaching and supervisory role—for resident physicians as they see patients in the outpatient clinic. Residents who have not yet attained licensure present their findings and we go to see the patient together. More advanced trainees present the finer points of their assessments and plans. For patients facing more complex situations, we take more time to discuss options for diagnosis and treatment. Mostly, we talk about evidence in the context of individual patients’ lives, conversations that soon alleviated the concerns I had of impending irrelevance. Which of several guidelines for blood pressure management should we apply? Under what circumstances would we prescribe both aspirin and an anticoagulant together? What about a high-dose opioid analgesic at the request of a new patient with no medical records? Which, if any, antibiotic is appropriate for a homebound patient with recurrent urinary tract infections who declines to be transported for an imaging study?

Over and over, evidence derived from carefully reviewed journal articles—the more openly available, the better—comes under the lens of each patient’s story, by contrast confidential and unique. This application of public, objective knowledge within the parameters of private, individual lives makes medicine a peculiar calling, neither art nor science exactly. During clinic, of course, we have little time to reflect on the quantitative roots and qualitative outcomes of our work; the schedule is busy. We do our best to identify the relevant evidence and work out a plan that makes practical sense for each patient. The role of occasional preceptor is not that of primary care physician—seeing patients in continuity over a long time—but this role does renew a sense of my experience, and my journal work, as relevant. Thus refreshed, I try to figure out why the electronic prescriptions I transmitted have twice failed to reach the patient’s pharmacy.

The future may not be what it was, but the need remains for sound evidence to support clinical practice. Continuing their involvement in the care of patients is an excellent idea for physicians who become journal editors, not only to apply their clinical training but also to promote mutual understanding between two distinct but interdependent professions. Clinic administrators and publishers of clinical journals should encourage such involvement to maintain the vital connection between “the literature” and the realities of medical practice, which should always inform an editor’s perspective on what is relevant.
